# The Risk Stratification of Papillary Thyroid Cancer With Bethesda Category III (Atypia of Undetermined Significance/Follicular Lesion of Undetermined Significance) by Thyroid Fine‐Needle Aspiration Could Be Assisted by Tumor Size for Precision Treatment

**DOI:** 10.3389/fendo.2022.822423

**Published:** 2022-02-07

**Authors:** Xiaojuan Zha, Zhenchun Miao, Xiu Huang, Xingchun Wang, Ruting Xie, Jiaoying Jin, Dajin Zou, Peng Yang, Yueye Huang

**Affiliations:** ^1^ Shanghai Center of Thyroid Diseases, Shanghai Tenth People’s Hospital, School of Medicine, Tongji University, Shanghai, China; ^2^ Department of Endocrinology and Metabolism, Shanghai Tenth People’s Hospital, School of Medicine, Tongji University, Shanghai, China; ^3^ Department of Pathology, Shanghai Tenth People’s Hospital, School of Medicine, Tongji University, Shanghai, China

**Keywords:** Bethesda category III, papillary thyroid cancer, fine‐needle aspiration, extra-thyroid extension, active surveillance

## Abstract

**Purpose:**

To investigate the clinical characteristics of papillary thyroid cancer (PTC) classified as Bethesda category III [atypia of undetermined significance (AUS)/follicular lesion of undetermined significance (FLUS)] by fine-needle aspiration (FNA) for precision treatment.

**Methods:**

A total of 1,739 patients diagnosed with Bethesda category III (AUS/FLUS) by FNA were investigated, and 290 patients diagnosed with PTC were analyzed.

**Results:**

The rate of papillary thyroid microcarcinoma (PTMC) was 82.1% (238/290). The rates of lymph node metastases were 44.9% (22/49) and 25.2% (56/222) for PTC and PTMC, respectively (p = 0.006). The rates of extra-thyroid extension were 46.2% (24/52) and 19.8% (47/237) (p < 0.001). Compared with PTMC, PTC had significantly higher odds ratios (ORs) of 3.41 (1.81–6.44, p < 0.001), 2.19 (1.16–4.13, p = 0.016), and 2.51 (1.29–4.88, p = 0.007) for extra-thyroid extension, multifocality, and lymph node metastases, respectively, after adjustment for age and gender. The larger size and BRAF V600E mutation had a robust synergistic effect for invasive features. The rates of lymph node metastases, multifocality, and extra-thyroid extension were significantly increased with larger sizes harboring BRAF V600E mutation. Compared with PTMC harboring wild type (WT)-BRAF, PTC harboring BRAF V600E mutation had adjusted higher ORs of 3.01 (1.26–8.68, p = 0.015), 3.20 (1.22–8.42, p = 0.018), and 5.62 (2.25–14.01, p < 0.001) for lymph node metastases, multifocality, and extra-thyroid extension, respectively.

**Conclusions:**

In this study, risk stratification was recommended for patients with Bethesda category III (AUS/FLUS) nodules with a size under 1 cm harboring WT-BRAF being regarded as low risk and should be recommended for active surveillance. Nodules with a size over 1 cm harboring WT-BRAF or those under 1 cm harboring BRAF V600E mutation could be regarded as moderate risk, and molecular testing should be recommended. However, those with a size over 1 cm harboring BRAF V600E mutation should be regarded as high risk, and a diagnostic surgery should be recommended.

## Introduction

Thyroid cancer (TC) was the most common endocrine malignancy, with a rapidly increasing number of cases over the past decades and 52,890 new cases in the United States in 2020 (1–4). TC was reported to be the fifth most common malignant tumor in women in 2020, with an incidence rate of approximately 4% (4). The increased rate can be predominantly attributed to the rapid development of various new imaging technologies (ultrasound (US), CT, and MRI) for the assessment of the thyroid gland. Moreover, environmental risk factors are also possible causes; the underlying causes of thyroid cancer incidence may be multifactorial (5, 6). Incidental thyroid nodules are found on 20% to 67% of US examinations (7). Fine-needle aspiration (FNA) played an important role in the management of the thyroid nodules by estimating the risk of malignancy and assisting the initial treatment of patients (8).

In 2009, The Bethesda System for Reporting Thyroid Cytopathology (TBSRTC) was released to refine cytologic definitions and improve clinical management for patients with thyroid nodules with surgery or surveillance (9). Based on the TBSRTC criteria, cytological diagnoses consist of six categories: I) non-diagnostic or unsatisfactory; II) benign; III) atypia of undetermined significance or follicular lesion of undetermined significance (AUS/FLUS); IV) follicular neoplasm or suspicious for a follicular neoplasm (FN/SFN); V) suspicious for malignancy; and VI) malignant (10). The “atypia of undetermined significance/follicular lesion of undetermined significance” (AUS/FLUS) category, known as Bethesda category III, has been ascribed to have a malignancy risk of 5%–15% (10). However, previous studies reported much higher malignancy rates in thyroid nodules classified as (AUS/FLUS) (11, 12), and the treatment for these cases remains controversial. The American Thyroid Association (ATA) guidelines recommended that repeat FNA or molecular testing should be applied to assist malignancy risk stratification instead of a strategy of either surveillance or diagnostic surgery for nodules with AUS/FLUS cytology (13). Sonographic features, patient preference, and feasibility should also be taken into consideration in treatment decision making (13). However, it is still challenging for clinicians to treat patients with Bethesda category III (AUS/FLUS), and the initial management requires further investigation.

The objective of this study was to describe the experience of a retrospective cohort of patients with Bethesda category III (AUS/FLUS) at Shanghai Center of Thyroid Diseases and diagnosed with papillary thyroid cancer (PTC) by surgery, as well as to investigate if there were any clinical characteristics that could assist to make risk stratification for Bethesda category III (AUS/FLUS).

## Materials and Methods

This retrospective study was approved by the Ethical Committee of the Shanghai Tenth People’s Hospital affiliated with Tongji University School of Medicine. Written informed consent was obtained from all patients (SHSY-IEC-4.1/21-33/01).

### Patients and Clinical–Pathological Data

Patients who underwent thyroidectomies or thyroid lobectomies at the Shanghai Tenth People’s Hospital from August 2015 to September 2020 were investigated. A total of 1,739 patients were diagnosed with Bethesda category III (AUS/FLUS) by FNA; 523 patients underwent thyroidectomy or thyroid lobectomy. The postoperative pathological results were 210 benign lesions and 23 other types of malignant tumors. In this study, 290 cases with PTC were analyzed. BRAF V600E mutation analysis was performed by the Sanger sequencing method in 287 postoperative tissue specimens at the pathology department of the university hospital.

### Statistical Analyses

Statistical analyses were performed with SPSS version 22.0 software (IBM Corporation, Armonk, NY, USA). Descriptive statistics were displayed as mean ± SD. Count data were expressed as number (n) and percentage (%). An independent-samples t-test was used to compare continuous data between two independent groups. The chi-squared test was used to analyze the categorical variables as appropriate. Univariate and multivariable logistic regressions were performed to analyze the correlation between lymph node metastasis, multifocality, extra-thyroid extension, and different tumor sizes combined with BRAF V600E mutation status. Odds ratios (ORs) with 95% CIs were calculated. A two-tailed p-value of less than 0.05 was considered statistically significant.

## Results

### Demographic and Clinical Characteristics

The clinical characteristics of all patients with PTC classified as Bethesda category III (AUS/FLUS) are shown in **Table 1**. The malignancy rate in thyroid nodules classified as Bethesda category III (AUS/FLUS) was 18.0% (313/1739). A total of 290 patients were diagnosed with PTC, and another malignant pathology was detected in 4.4% (23/523) and benign nodules in 40.2% (210/523) of patients. The cohort of 290 patients with PTC (227 females and 63 males) had a median age of 48 years (interquartile range [IQR], 21–77) and was investigated retrospectively. Papillary thyroid microcarcinoma (PTMC) was more common than PTC, with a rate of 82.1% (238/290) (**Table 2**). The rates of lymph node metastasis were 44.9% (22/49) and 25.2% (56/222) for PTC and PTMC (p = 0.006), respectively. The rates of BRAF V600E mutation for PTMC and PTC were 64.6% (153/237) and 64.0% (32/50), respectively (**Table 2**). The rates of extra-thyroid extension were 46.2% (24/52) and 19.8% (47/237) for PTC and PTMC, respectively (p < 0.001). Compared with PTMC, PTC had higher crude ORs of 3.47 (1.84–6.52, p < 0.001), 2.20 (1.17–4.13, p = 0.014), and 2.42 (1.28–4.58, p = 0.007) for extra-thyroid extension, multifocality, and lymph node metastasis, respectively. After adjustment for age and gender, PTC had higher ORs of 3.41 (1.81–6.44, p < 0.001), 2.19 (1.16–4.13, p = 0.016), and 2.51 (1.29–4.88, p = 0.007) for extra-thyroid extension, multifocality, and lymph node metastasis (**Table 3**).

**Table 1:** The demographic and clinical characteristics of PTC patients by cytology type (n = 290).

Variants	AUS (N = 229)	FLUS and AUS/FLUS (N = 61)	p
Age (year)	47.68 ± 12.39	49.90 ± 12.19	0.212
Gender			0.794
Male	49 (21.4%)	14 (23.0%)	
Female	180 (78.6%)	47 (77.0%)	
Tumor size (cm)			0.981
≤1	188 (82.1%)	50 (82.0%)	
>1	41 (17.9%)	11 (18.0%)	
Tumor size (cm)	0.68 ± 0.54	0.69 ± 0.53	0.968
Focality			0.360
Unifocality	171 (74.7%)	42 (68.9%)	
Multifocality	58 (25.3%)	19 (31.1%)	
Extra-thyroid extension			0.500
No	174 (76.3%)	44 (72.1%)	
Yes	54 (23.7%)	17 (27.9%)	
Surgery			0.790
Total thyroidectomy	158 (69.0%)	41 (67.2%)	
Thyroid lobectomy	71 (31.0%)	20 (32.8%)	
BRAF V600E			0.838
Wild type	81 (35.8%)	21 (34.4%)	
Mutation	145 (64.2%)	40 (65.6%)	
Lymph node metastasis			0.463
No	148 (70.1%)	45 (75.0%)	
Yes	63 (29.9%)	15 (25.0%)	
I-131 therapy			0.945
No	211 (92.1%)	57 (93.4%)	
Yes	18 (7.9%)	4 (6.6%)	

One patient with the status of extra-thyroid extension remaining unknown was excluded. Nineteen patients with the status of lymph node metastasis remaining unknown were excluded. Three patients with the BRAF V600E mutation status remaining unknown were excluded.

PTC, papillary thyroid cancer; AUS, atypia of undetermined significance; FLUS, follicular lesion of undetermined significance.

**Table 2:** The demographic and clinical characteristics of PTC patients by tumor size (n = 290).

	PTMC (N = 238)	PTC (N = 52)	p
Age (year)	47.93 ± 12.02	49.12 ± 13.93	0.533
Gender			0.527
Male	50 (21.0%)	13 (25.0%)	
Female	188 (79.0%)	39 (75.0%)	
Cytology			0.981
AUS	188 (79.0%)	41 (78.8%)	
FLUS and AUS/FLUS	50 (21.0%)	11 (21.2%)	
Focality			0.013
Unifocality	182 (76.5%)	31 (59.6%)	
Multifocality	56 (23.5%)	21 (40.4%)	
Extra-thyroid extension			<0.001
No	190 (80.2%)	28 (53.8%)	
Yes	47 (19.8%)	24 (46.2%)	
Surgery			0.274
Total thyroidectomy	160 (67.2%)	39 (75.0%)	
Thyroid lobectomy	78 (32.8%)	13 (25.0%)	
BRAF V600E			0.940
Wild type	84 (35.4%)	18 (36.0%)	
Mutation	153 (64.6%)	32 (64.0%)	
Lymph node metastasis			0.006
No	166 (74.8%)	27 (55.1%)	
Yes	56 (25.2%)	22 (44.9%)	
I-131 therapy			0.001
No	226 (95.0%)	42 (80.8%)	
Yes	12 (5.0%)	10 (19.2%)	

One patient with the status of extra-thyroid extension remaining unknown was excluded. Nineteen patients with the status of lymph node metastasis remaining unknown were excluded. Three patients with the status of BRAF V600E mutation status remaining unknown were excluded.

PTMC, papillary thyroid microcarcinoma; PTC, papillary thyroid cancer; AUS, atypia of undetermined significance; FLUS, follicular lesion of undetermined significance.

**Table 3:** Logistic regression analysis of factors of PTC with different tumor sizes.

Group	Unadjusted			Adjusted^a^	
	n/N (%)	OR (95% CI)	p	OR (95% CI)	p
Extra-thyroid extension^b^	71/289 (24.6)				
≤1 cm	47/237 (19.8)	Ref.	Ref.	Ref.	Ref.
>1 cm	24/52 (46.2)	3.47 (1.84–6.52)	<0.001	3.41 (1.81–6.44)	<0.001
Multifocality	77/290 (26.6)				
≤1 cm	56/238 (23.5)	Ref.	Ref.	Ref.	Ref.
>1 cm	21/52 (40.4)	2.20 (1.17–4.13)	0.014	2.19 (1.16–4.13)	0.016
Lymph node metastasis	78/271 (28.8)				
≤1 cm	56/222 (25.2)	Ref.	Ref.	Ref.	Ref.
>1 cm	22/49 (44.9)	2.42 (1.28–4.58)	0.007	2.51 (1.29–4.88)	0.007

OR, odds ratio; PTC, papillary thyroid cancer.

a Adjusted for age and gender.

b One patient with the status of extra-thyroid extension remaining unknown was excluded. Nineteen patients with the status of lymph node metastasis remaining unknown were excluded.

### The Synergistic Effect of Tumor Size and BRAF V600E Mutation Status in Papillary Thyroid Cancer With Atypia of Undetermined Significance/Follicular Lesion of Undetermined Significance by Fine-Needle Aspiration

The effects of risk stratification by tumor size and BRAF V600E mutation are shown in **Table 4**. The rates of lymph node metastasis were 21.4% (18/84), 24.8% (38/153), 38.9% (7/18), and 43.8% (14/32) for PTMC harboring wild type (WT)-BRAF, PTMC harboring BRAF V600E mutation, PTC harboring WT-BRAF, and PTC harboring BRAF V600E mutation, respectively. The rates of multifocality were 15.5% (13/84), 28.1% (43/153), 44.4% (8/18), and 37.5% (12/32) for PTMC harboring WT-BRAF, PTMC harboring BRAF V600E mutation, PTC harboring WT-BRAF, and PTC harboring BRAF V600E mutation, respectively. The rates of extra-thyroid extension were 17.9% (15/84), 20.9% (32/153), 33.3% (6/18), and 56.3% (18/32) for PTMC harboring WT-BRAF, PTMC harboring BRAF V600E mutation, PTC harboring WT-BRAF, and PTC harboring BRAF V600E mutation, respectively. PTC harboring BRAF V600E mutation had the highest rates of lymph node metastases and extra-thyroid extension, while PTMC harboring WT-BRAF had the lowest rates. Compared with PTMC harboring WT-BRAF, PTC harboring BRAF V600E mutation had a crude OR of 2.55 (1.06–6.12, p = 0.036) for lymph node metastasis and remained significant after adjustment for age and gender [3.01 (1.26–8.68), p = 0.015]. Compared with the PTMC harboring WT-BRAF group, PTC harboring BRAF V600E mutation had a crude OR of 5.83 (2.38–14.26, p < 0.001) for extra-thyroid extension and remained significant after adjustment for age and gender [5.62 (2.25–14.01), p < 0.001]. Compared with the PTMC harboring WT-BRAF group, the other three groups had crude ORs of 2.14 (1.07–4.25, p = 0.031), 4.37 (1.45–13.15, p = 0.009), and 3.28 (1.30–8.29, p = 0.012) for multifocality, and all remained significant after adjustment for age and gender (**Table 5**).

**Table 4:** The clinical characteristics of PTC patients by tumor size and BRAF V600E mutation status (n = 287).

Variants	≤1cm+WT-BRAF (N = 84)	≤1cm+BRAF Mutation (N = 153)	>1cm+WT-BRAF (N = 18)	>1cm+BRAF Mutation (N = 32)
Lymph node metastasis				
No	59 (70.2%)	106 (69.3%)	9 (50.0%)	18 (56.3%)
Yes	18 (21.4%)	38 (24.8%)	7 (38.9%)	14 (43.8%)
Unknown	7 (8.3%)	9 (5.9%)	2 (11.1%)	0 (0%)
Multifocality				
No	71 (84.5%)	110 (71.9%)	10 (55.6%)	20 (62.5%)
Yes	13 (15.5%)	43 (28.1%)	8 (44.4%)	12 (37.5%)
Extra-thyroid extension				
No	68 (80.9%)	121 (79.1%)	12 (66.7%)	14 (43.8%)
Yes	15 (17.9%)	32 (20.9%)	6 (33.3%)	18 (56.3%)
Unknown	1 (1.2%)	0 (0%)	0 (0%)	0 (0%)

One patient with the status of extra-thyroid extension, eighteen patients with the status of lymph node metastasis, and another three patients with the status of BRAF V600E mutation remaining unknown were excluded.

PTC, papillary thyroid cancer.

**Table 5:** The relationship of clinical invasive features and synergistic effect of tumor size and BRAF *V600E* mutation status by logistic regression analysis.

Group	Unadjusted			Adjusted^a^	
	n/N (%)	OR (95% CI)	p	OR (95% CI)	p
Lymph node metastasis					
≤1cm+WT-BRAF	18 (21.4)	Ref.	Ref.	Ref.	Ref.
≤1cm+BRAF mutation	38 (24.8)	1.18 (0.62–2.24)	0.624	1.24 (0.63–2.43)	0.530
>1cm+WT-BRAF	7 (38.9)	2.55 (0.83–7.81)	0.101	2.14 (0.67–6.85)	0.202
>1cm+BRAF mutation	14 (43.8)	2.55 (1.06–6.12)	0.036	3.01 (1.26–8.68)	0.015
Multifocality					
≤1cm+WT-BRAF	13 (15.5)	Ref.	Ref.	Ref.	Ref.
≤1cm+BRAF mutation	43 (28.1)	2.14 (1.07–4.25)	0.031	2.17 (1.08–4.34)	0.029
>1cm+WT-BRAF	8 (44.4)	4.37 (1.45–13.15)	0.009	4.84 (1.56–15.06)	0.006
>1cm+BRAF mutation	12 (37.5)	3.28 (1.30–8.29)	0.012	3.20 (1.22–8.42)	0.018
Extra-thyroid extension					
≤1cm+WT-BRAF	15 (17.9)	Ref.	Ref.	Ref.	Ref.
≤1cm+BRAF mutation	32 (20.9)	1.20 (0.61–2.37)	0.602	1.13 (0.57–2.26)	0.724
>1cm+WT-BRAF	6 (33.3)	2.27 (0.73–7.01)	0.155	2.01 (0.62–6.48)	0.244
>1cm+BRAF mutation	18 (56.3)	5.83 (2.38–14.26)	<0.001	5.62 (2.25–14.01)	<0.001

^a^Adjusted for age and gender.

OR, odds ratio; PTC, papillary thyroid cancer.

## Discussion

The ATA guidelines recommend that repeat FNA or molecular testing should be applied to assess malignancy risk stratification instead of surveillance or diagnostic surgery for nodules with AUS/FLUS cytology (13). Patients diagnosed with AUS/FLUS by FNA were generally considered to be at low risk and usually have an excellent prognosis. It is still necessary to perform risk stratification to determine the intrinsic risk for clinical aggressiveness and therefore favor more aggressive treatments. Conversely, intrinsically low-risk AUS/FLUS nodules or PTMCs should be treated with surveillance and should not be overtreated.

In this study, we investigated the clinical aggressiveness of PTC with AUS/FLUS by FNA before surgery, to determine the clinical markers to facilitate risk stratification in patients with AUS/FLUS nodules. In accordance with the diagnostic criteria given by the Japan Society of Ultrasonics in Medicine (JSUM), nodules that are 5.1–10.0 mm in diameter that are strongly suspicious for thyroid carcinoma should be recommended for FNA (14). Korean studies have shown that 0.6- to 0.9-cm nodules could be indicated for FNA when they have highly suspicious US features (15). Generally, nodules larger than 5 mm in diameter with US findings of signs of malignancy are recommended for FNA in China. Our data indicated that PTMCs are much more common than PTCs in patients with AUS/FLUS nodules. In addition, patients with a tumor size over 1 cm were more likely to have extra-thyroid extension. A cutoff value of 1 cm for tumor size could clearly allow for distinction between high- and low-risk groups. Furthermore, BRAF V600E mutation is important for risk stratification. Therefore, we concluded that a combination of tumor size and BRAF mutation could clearly differentiate AUS/FLUS into three categories with varying risk for more precise management of Bethesda category III (AUS/FLUS) lesions.

The TBSRTC was developed in 2007 and is widely accepted today (9). The National Cancer Institute convened a conference to define cytology terminology for thyroid nodules that have a risk for malignancy (9). The Bethesda System has greatly facilitated the standardization of FNA reporting. It has enabled more precise decision making when patients are recommended for surgery (16). Lesions with non-diagnostic and benign cytology should be suggested for active surveillance (AS), while operation may be recommended for malignancies (13). However, the management for Bethesda category III (AUS/FLUS) remains controversial. Based on ATA guidelines, the nodules classified as Bethesda category III (AUS/FLUS) are recommended for repeat FNAs or molecular testing to assess malignancy risk, and a panel of mutations (BRAF, NRAS, HRAS, KRAS, RET/PTC1, RET/PTC3, and PAX8/PPARγ) or 167 Gene Expression Classifier (GEC) can be recommended for precise molecular testing before deciding between surveillance and diagnostic surgery (13). The three isoforms of RAS (HRAS, NRAS, and KRAS) mutations are the second most common mutation encountered in thyroid carcinomas. It was found in about 40% of follicular variant papillary thyroid carcinomas (fvPTC) and 10% in classical PTCs (cPTCs). RET/PTC rearrangements were seen in about 10%–20% of papillary thyroid carcinomas, RET/PTC1 was associated with a more indolent behavior, and RET/PTC3 mutations were related to more aggressive behavior. They could not help distinguish benign nodules from malignant ones (17, 18). PAX8/PPARγ rearrangements were detected in about 2%–10% of follicular adenomas, as in the fvPTC. In addition, about 0%–1% of PTCs had PAX8/PPARγ translocations (18). BRAF V600E mutation and RET/PCT rearrangements showed high specificity and might help diagnose malignancy in mutated thyroid nodules (19). Molecular tests in thyroid FNA specimens had already shown obvious clinical utility in improving the preoperative diagnosis of thyroid cancer and had been helpful in the risk stratification of Bethesda category III cytological results (20). Guo et al. reported that BRAF V600E and RAS mutations and RET/PTC rearrangements were found in 65.1%, 0%, and 1.6% of PTCs, respectively (21).

The BRAF V600E mutation has drawn particular attention in thyroid FNA. Muzza et al. recommended two different categories of molecular tests for reducing the avoidable treatment of benign nodules and optimizing surgical management. Two different categories of molecular tests can be thought of as “rule-out” methods or “rule-in” tests for malignancy. The Afirma GEC of the “rule-out” methods aims to predict benign thyroid nodules and prevent overtreatment, while the ThyroSeq assay of “rule-in” tests could detect malignancy and optimize surgical management (22–24). The Afirma GEC was established as a good “rule-out” test and the ThyroSeq assay as a good “rule-in” test (17). Molecular tests can rule in cancer for indeterminate thyroid nodules with highly specific mutations for cancer, such as BRAF and RET/PTC. In selected cases, molecular testing can rule out thyroid cancer including a low prevalence of cancer without high-risk history, physical features, or US features (25). Ooi et al. demonstrated that BRAF mutational analyses are used in Asian centers especially in China and Korea, while gene panels and gene expression classifiers are used in the United States (26). Sun et al. reported that the synergism of BRAF V600E testing combined with FNA cytology could increase the diagnostic sensitivity and decrease the false-negative rate by 87.4% and 5.2%, respectively. In AUS/FLUS, the sensitivity rate of BRAF V600E was 40.1%, while the specificity rate was 99.5% (27). Cohen et al. investigated 231 patients and found that patients with BRAF V600E and RET mutations in thyroid nodules with AUS/FLUS features detected by FNA had a 100% risk of malignancy (28). Yin et al. reported that the diagnostic rates of AUS/FLUS could be reduced from 11.59% to 8.42% by BRAF V600E detection, while all AUS/FLUS cases harboring BRAF V600E were diagnosed as PTC from 10.92% to 7.93% in 2017. Therefore, the results of this study could help clinicians devise treatment strategies and determine patient prognosis effectively (29). Suh et al. investigated 667 patients with AUS/FLUS who underwent surgery and reported that the BRAF V600E mutation combined with K-TIRADS 5 could predict malignancy and reduce unnecessary surgeries for AUS/FLUS nodules (30).

Our data demonstrated that the nodule size and BRAF V600E status could be used to clearly divide AUS/FLUS into low-, moderate- and high-risk categories. Nodules under 1 cm harboring WT-BRAF were considered low risk, and once-a-year surveillance was recommended. Nodules under 1 cm harboring BRAF V600E mutation or nodules over 1 cm harboring WT-BRAF were considered a moderate risk, and molecular testing was recommended. If negative, surveillance once every 6 months should be applied, while diagnostic surgery could be recommended if the nodule tests positive. Nodules over 1 cm harboring BRAF V600E mutation were considered as high risk, and a diagnostic surgery is recommended.

In the 2017 TBSRTC, the malignancy rate for Bethesda category III (AUS/FLUS) was revised, and it was 10%–30% higher than originally estimated and reported in 2009 (10). The management of these patients included FNA repeating, molecular testing, or lobectomy (10). This resulted in further questions being raised regarding the appropriate testing to be used in further investigations. Herein, we recommended that AUS/FLUS nodules under 1 cm should receive testing for the BRAF V600E mutation and that WT-BRAF suggested a very low risk of the nodules, with surveillance once in a year considered to be adequate. Although there were some patients who had a high intrinsic risk for being a PTMC, surveillance was still appropriate for them. In the two past decades, the incidence of thyroid cancer has increased significantly in the United States (31), Korea (32), and China (33), while thyroid cancer-specific mortality has not increased. The overdiagnosis and overtreatment of PTMCs are common problems worldwide (31, 32). The initiation of AS for low-risk PTC was developed in Japan, in the Kuma Hospital, which conducted the first clinical trial; and the Cancer Institute Hospital in Tokyo started the second trial 2 years later (34). After a 10-year observation period in the Kuma Hospital with 1,235 patients, only 8% and 3.8% of the patients with PTMC showed size enlargement by ≥3 mm and novel appearance of node metastasis, respectively. In the Kuma Hospital, the 974 patients who underwent immediate surgery had significantly higher incidences of adverse events than the 1,179 patients who chose AS. Oda et al. proposed that the immediate-surgery group presented with a higher incidence of permanent vocal cord paralysis (0.2%) and permanent hypoparathyroidism (1.6%) compared with the AS group (35). In the AS group, the incidences of tumor growth and lymph node metastases were similar to those in other trials. Smulever et al. suggested that AS could be applied for low-risk PTCs because of unfavorable postoperative complications (36). The cost of treatment was particularly important. In the 10-year study, the total cost of immediate surgery was 4.1 times higher when compared with AS, including the cost of postoperative care. In the Cancer Institute Hospital trial, the rate of tumor enlargement and new lymph node metastasis for AS was 7% and 1%, respectively. In the two previous trials, none of the patients had obvious relapses or death due to PTC (37). For low-risk patients with PTMC, AS became the first-line treatment (37). Miyauchi et al. demonstrated that PTMCs were less likely to grow in older patients (≥60 years) in contrast to PTCs. Therefore, AS was recommended for elderly patients with low-risk PTMCs (37). In 2017, Tuttle et al. published the first research on AS for low-risk PTMC outside of Japan where they investigated 291 patients and found 3.8% showed growth in tumor diameter of 3 mm or more, and no patients had regional or distant metastasis during the surveillance (38). Ito et al. proposed that the rate of growth of PTMCs during pregnancy and delivery was low at 8%, and there was no lymph node metastasis. Therefore, AS was also recommended during pregnancy (39). Based on previous studies, AS was recommended to be the first-line management for low-risk PTMCs. It was also reasonable for low-risk AUS/FLUS nodules to be kept under surveillance, instead of performing repeat FNAs or molecular testing for anything other than a BRAF V600E mutation.

However, previous studies reported much higher malignancy rates in thyroid nodules classified as AUS/FLUS (11, 12). In this study, given the high malignancy rate of PTC (290/523, 55.4%), there were intrinsic high-risk PTCs that were contraindications for AS. Our data demonstrated that patients with tumors over 1 cm had a much higher OR of extra-thyroid extension. Huang et al. reported that the BRAF V600E mutation could help clearly differentiate low-risk solitary intra-thyroidal PTC into low- and high-risk categories (1). Kim et al. investigated 743 patients treated using a total thyroidectomy for PTMC (584 females and 159 males), and results showed that the rate of tumor recurrence was lower in BRAF V600E-negative (6.4%) than BRAF V600E-positive patients (10.8%) (40). Among the low-risk PTMC patients, recurrences were 1.3% for BRAF V600E-negative patients and 4.3% for BRAF V600E-positive patients. Therefore, the recurrence risk of PTMC could be identified by BRAF V600E mutation status, especially for low-risk PTMCs. In low-risk PTMC with WT-BRAF, conservative AS was suitable for management. Due to the increased recurrence risk and other adverse effects, including extra-thyroid extension, AS was still uncertain for BRAF V600E mutation patients with PTMC so far (40). In our study, patients with PTC tumors over 1 cm harboring BRAF V600E mutation had higher rates of lymph node metastasis, multifocality, and extra-thyroid extension. Based on our results and previous studies, the synergistic effect of nodule size and BRAF V600E mutation status has increased the possibility of predicting the risk of malignancy of PTC with AUS/FLUS nodules.

## Conclusions

In conclusion, it is necessary to improve risk stratification for AUS/FLUS nodules. A combination of nodule size and BRAF V600E mutation status could help clearly differentiate AUS/FLUS into three categories with different risks for malignancy to help manage patients with AUS/FLUS nodules more precisely. An AUS/FLUS nodule with a size under 1 cm harboring WT-BRAF could be recommended for AS, those with a size over 1 cm harboring WT-BRAF or size under 1 cm harboring BRAF V600E mutation could be recommended for repeat FNAs or molecular testing, and AUS/FLUS nodules with size over 1 cm and BRAF V600E mutation could receive aggressive treatment, and a diagnostic surgery may be recommended.

## Data Availability Statement

The original contributions presented in the study are included in the article/supplementary material. Further inquiries can be directed to the corresponding author.

## Ethics Statement

The studies involving human participants were reviewed and approved by the Ethical Committee of the Shanghai Tenth People’s Hospital affiliated with Tongji University School of Medicine. Written informed consent was obtained from all patients (SHSY-IEC-4.1/21-33/01). The patients/participants provided their written informed consent to participate in this study.

## Author Contributions

Conception and design: XZ and YH. Acquisition, statistical analysis, or interpretation of the data: all authors. Drafting of the manuscript: all authors. All authors reviewed and approved the final version of the manuscript.

## Funding

This study was funded by the National Natural Science Foundation of China (grant number 81902716) and Shanghai Pujiang Program (grant number 2019PJD040).

## Conflict of Interest

The authors declare that the research was conducted in the absence of any commercial or financial relationships that could be construed as a potential conflict of interest.

## Publisher’s Note

All claims expressed in this article are solely those of the authors and do not necessarily represent those of their affiliated organizations, or those of the publisher, the editors and the reviewers. Any product that may be evaluated in this article, or claim that may be made by its manufacturer, is not guaranteed or endorsed by the publisher.
