# Corrigendum: The Risk Stratification of Papillary Thyroid Cancer With Bethesda Category III (Atypia of Undetermined Significance/Follicular Lesion of Undetermined Significance) by Thyroid Fine‐Needle Aspiration Could Be Assisted by Tumor Size for Precision Treatment

**DOI:** 10.3389/fendo.2022.911963

**Published:** 2022-05-18

**Authors:** Xiaojuan Zha, Zhenchun Miao, Xiu Huang, Xingchun Wang, Ruting Xie, Jiaoying Jin, Dajin Zou, Peng Yang, Yueye Huang

**Affiliations:** ^1^ Shanghai Center of Thyroid Diseases, Shanghai Tenth People’s Hospital, School of Medicine, Tongji University, Shanghai, China; ^2^ Department of Endocrinology and Metabolism, Shanghai Tenth People’s Hospital, School of Medicine, Tongji University, Shanghai, China; ^3^ Department of Pathology, Shanghai Tenth People’s Hospital, School of Medicine, Tongji University, Shanghai, China

**Keywords:** Bethesda category III, papillary thyroid cancer, fine‐needle aspiration, extra-thyroid extension, active surveillance

In the originally published article, there were errors each of the author affiliations.

Affiliation 1 should have been presented as “Shanghai Center of Thyroid Diseases, Shanghai Tenth People’s Hospital, School of Medicine, Tongji University, Shanghai, China”, and not as “Shanghai Center of Thyroid Diseases, Shanghai, China”.

Affiliation 2 should have been presented as “Department of Endocrinology and Metabolism, Shanghai Tenth People’s Hospital, School of Medicine, Tongji University, Shanghai, China”, and not as “Department of Endocrinology and Metabolism, Shanghai Tenth People’s Hospital, Tongji University School of Medicine, Shanghai, China”

Affiliation 3 should have been presented as “Department of Pathology, Shanghai Tenth People’s Hospital, School of Medicine, Tongji University, Shanghai, China”, and not as Department of Pathology, Shanghai Tenth People’s Hospital, Tongji University School of Medicine, Shanghai, China”.

The authors apologize for these errors and state that they do not change the scientific conclusions of the article in any way. The original article has been updated.

## Publisher’s Note

All claims expressed in this article are solely those of the authors and do not necessarily represent those of their affiliated organizations, or those of the publisher, the editors and the reviewers. Any product that may be evaluated in this article, or claim that may be made by its manufacturer, is not guaranteed or endorsed by the publisher.

